# *Geosesarma sodalis*, a new species of vampire crab (Crustacea, Brachyura, Sesarmidae) from a limestone cave in central Sarawak, Malaysia

**DOI:** 10.3897/zookeys.1031.63134

**Published:** 2021-04-15

**Authors:** Peter K.L. Ng

**Affiliations:** 1 Lee Kong Chian Natural History Museum, Faculty of Science, National University of Singapore, Kent Ridge, Singapore 119260, Republic of Singapore National University of Singapore Singapore Singapore

**Keywords:** Borneo, cavernicolous, description, *
Geosesarma
*, karst, new taxon, taxonomy

## Abstract

A new species of semi-terrestrial crab of the genus *Geosesarma* (Sesarmidae) is described from a limestone cave in central Sarawak, Malaysian Borneo. *Geosesarma sodalis***sp. nov.** is characterised by its quadrate carapace, absence of a flagellum on the exopod of the third maxilliped, presence of 10 or 11 sharp tubercles on the dactylus of the chela and a diagnostic male first gonopod structure. This is the sixth species of *Geosesarma* reported from Sarawak, and the first member of the genus collected from inside caves.

## Introduction

In 2005, Rob Stuebing passed the author several brachyuran crabs he collected while surveying limestone caves in the Bintulu area in central Sarawak. The material included a new species of a cavernicolous gecarcinucid, and in 2006, fresh surveys were made in the caves to obtain more specimens. This new material formed the basis for the description of a new species of *Arachnothelphusa* Ng, 1991, by Grinang and Ng (2021).

Among the original 2005 material collected by Stuebing was a specimen of *Geosesarma* De Man, 1892 (Sesarmidae). Examination of the specimen showed it to be a new species, here named *Geosesarma sodalis* sp. nov. This is also the first record of a *Geosesarma* from inside caves. *Geosesarma* are often called vampire crabs because many species have bright yellow eyes in life (see Ng et al. 2015; Ng 2017). *Geosesarma* is a large genus, with 67 species known from Southeast and East Asia, the Andaman Islands, Papua New Guinea, and the Solomon Islands (Ng et al. 2008; Ng and Wowor 2019; Shy and Ng 2019; Naruse and Ng 2020).

## Material and methods

Measurements provided are the carapace width and length. The terminology used in this paper follows Ng et al. (2008) and Davie et al. (2015). The abbreviations G1 and G2 are used for the male first and second gonopods, respectively. The type specimen is deposited in the Zoological Reference Collection (**ZRC**) of the Lee Kong Chian Natural History Museum, National University of Singapore.

## Systematic accounts

### Family Sesarmidae Dana, 1851

#### Genus Geosesarma

Taxon classificationAnimaliaDecapodaSesarmidae

De Man, 1892

A145B6CF-9696-57B9-8F7E-EDDB72E402FB

##### Type species.

Sesarma (Geosesarma) nodulifera De Man, 1892; subsequent designation by Serène and Soh (1970).

#### Geosesarma sodalis sp. nov.

Taxon classificationAnimaliaDecapodaSesarmidae

62B0437F-0230-5D64-A5CD-428E177663A1

http://zoobank.org/69A4BE4D-8B0B-4243-9B2D-BA1D559A2C28

Figures 1 , 2 , 3

##### Material examined.

***Holotype*:** male (10.1 × 9.8 mm) (ZRC 2020.0413), limestone cave, Bukit Sarang, Bintulu, Sarawak, Malaysia, coll. Stuebing RB, early 2005.

##### Diagnosis.

**Figure 1.**
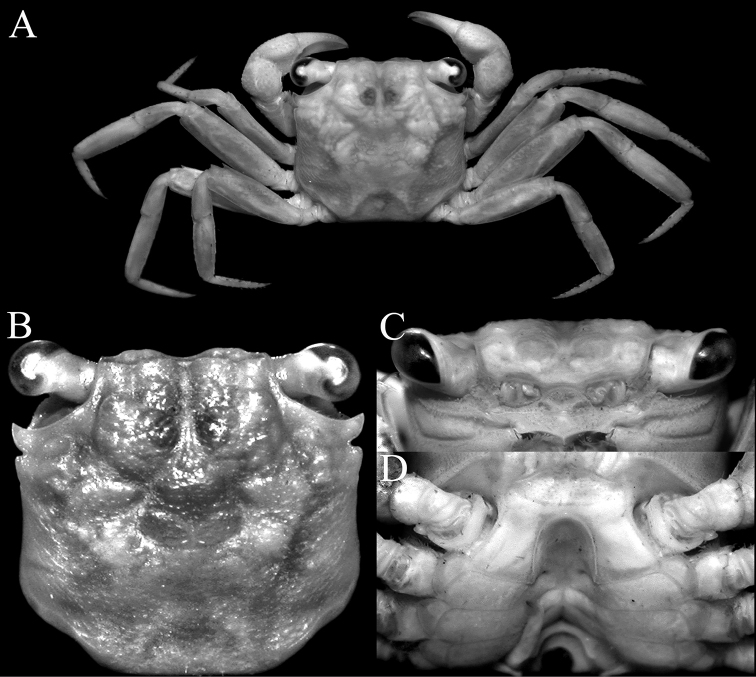
*Geosesarma sodalis* sp. nov., holotype male (10.1 × 9.8 mm) (ZRC 2020.0413), Sarawak **A** overall dorsal view **B** dorsal view of carapace **C** frontal view of cephalothorax **D** anterior thoracic sternites and sternopleonal cavity.

**Figure 2.**
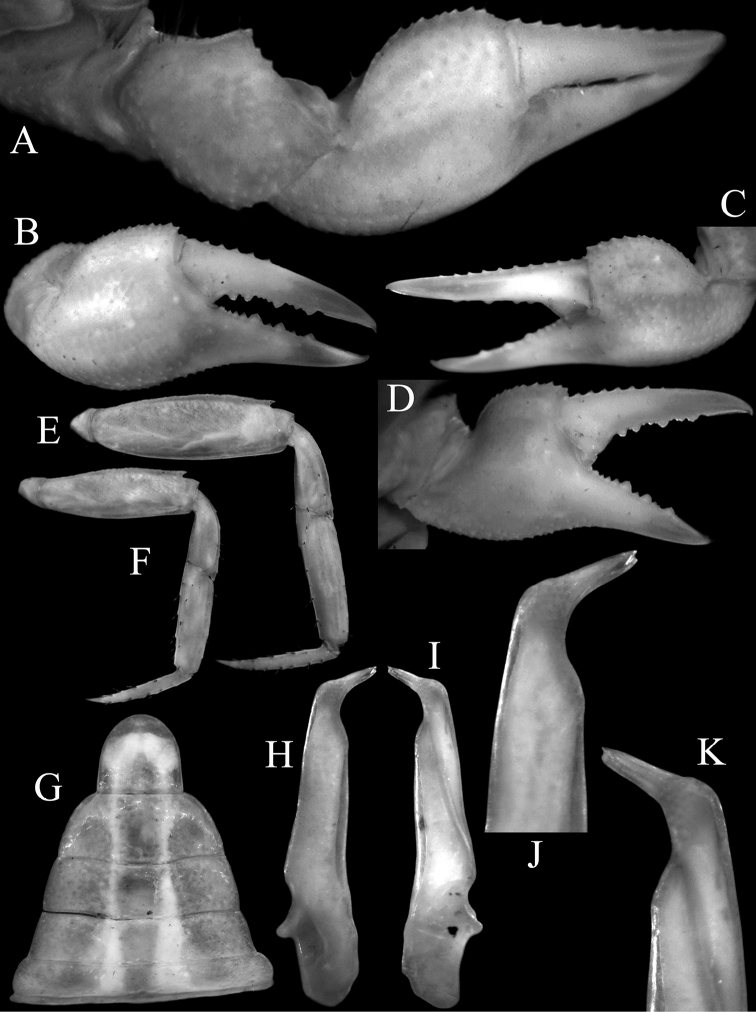
*Geosesarma sodalis* sp. nov., holotype male (10.1 × 9.8 mm) (ZRC 2020.0413), Sarawak **A** dorsal view of right cheliped **B** outer view of right chela **C** subdorsal view of left chela **D** inner view of right chela **E** right third ambulatory leg **F** right fourth ambulatory leg **G** pleonal somites 2–6 and telson **H** left G1 (ventral view) **I** left G1 (ventral view) **J** distal part of left G1 (ventral view) **K** distal part of left G1 (ventral view).

**Figure 3.**
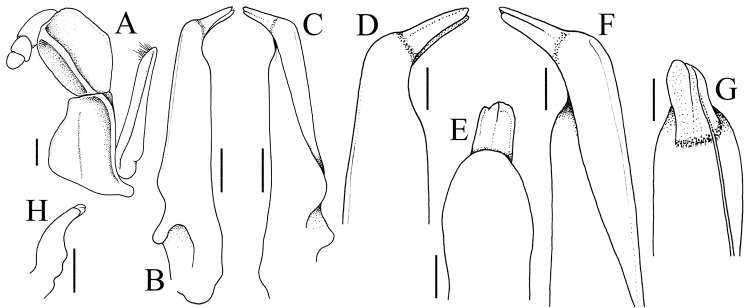
*Geosesarma sodalis* sp. nov., holotype male (10.1 × 9.8 mm) (ZRC 2020.0413), Sarawak **A** left third maxilliped (setae denuded) **B** left G1 (ventral view) **C** left G1 (ventral view) **D** distal part of left G1 (ventral view) **E** distal part of left G1 (distomesial view) **F** distal part of left G1 (ventral view) **G** distal part of left G1 (ventromesial view) **H** left G2. Scales bars: 0.5 mm (**A–C, H**); 0.25 mm (**D–G**).

Carapace quadrate, slightly wider than long, width to length ratio 1.03, lateral margins gently concave, subparallel (Fig. 1A, B); dorsal surfaces with well-defined regions, anterior half with low granules, posterior half almost smooth (Fig. 1A, B); frontal margin distinctly deflexed, frontal lobes broad, with truncated margins in dorsal view, separated by wide shallow median concavity; postfrontal and postorbital cristae sharp, distinct (Fig. 1A–C); external orbital angle triangular, directed obliquely anteriorly, extending just beyond lateral carapace margins, outer lateral margin convex; separated from first epibranchial tooth by deep V-shaped cleft; first epibranchial tooth distinct, second epibranchial tooth visible only as low lobe, barely separated from first tooth by shallow concavity (Fig. 1A, B); merus of third maxilliped subovate; exopod slender, flagellum absent (Fig. 3A); outer surfaces of palm of chela covered with small rounded granules, inner surface without transverse ridge; fingers longer than palm, dorsal margin of dactylus with 10 or 11 sharp, anteriorly directed sharp tubercles (Fig. 2A–D); ambulatory merus with sharp subdistal spine on dorsal margin, surface weakly rugose, propodus slender, relatively long (Figs 1A, 2E, F); pleon triangular; somite 3 widest, somite 6 with lateral margins gently convex; telson triangular, longer than broad, lateral margins gently convex (Fig. 2G); G1 relatively slender, proximal, distal part bent at angle of ca. 45° along longitudinal axis, subdistal part of outer margin gently angular with shelf-like feature (Figs 2H–K, 3B–D, F), distal part elongate, tapering in lateral view, spatuliform in marginal view, with small submedian cleft at tip when viewed mesially (Fig. 3E, G).

##### Colour.

Not known.

##### Females.

Not known.

##### Etymology.

The name is derived from the Latin noun for comradeship; alluding to the deep friendship the author has had over the last 30 years with the collector, Rob Stuebing, who has collected many interesting species for him.

##### Remarks.

The island of Borneo has 13 known species of *Geosesarma*, all of which are endemic to the island. Five species occur in the state of Sarawak (Ng and Grinang 2018; Ng and Ng 2019). One group of *Geosesarma* species is characterised by their relatively quadrate carapace, presence of a row to sharp tubercles on the dorsal margin of the cheliped dactylus, absence of a flagellum on the third maxilliped exopod, and a relatively stout G1 with a tapering corneous distal part (in lateral view). In Borneo, the species in this group are *G. gracillimum* (De Man, 1902), *G. sabanus* Ng, 1992, *G. aurantium* Ng, 1995, *G. katibas* Ng, 1995, *G. danumense* Ng, 2002, *G. bau* Ng & Grinang, 2004, *G. ambawang* Ng, 2015, *G. pontianak* Ng, 2015, *G. larsi* Ng & Grinang, 2018, and *G. spectrum* Ng & Ng, 2019.

Five of the species in this group are present in Sarawak and Brunei: *G. gracillimum*, *G. katibas*, *G. bau*, *G. larsi*, and *G. sodalis* sp. nov. Compared to *G. gracillimum*, the carapace of *G. sodalis* sp. nov. is more quadrate with the lateral margins subparallel (Fig. 1A, b) (versus gently diverging in *G. gracillimum*; see Ng 2015: fig. 14A, B; Ng and Ng 2015: fig. 5F). The G1 of *G. sodalis* sp. nov. (Figs 2H, I, 3B, C) is distinct in that it is proportionately more slender than those of *G. gracillimum*, *G. katibas*, and *G. larsi* (cf. Ng 1995: fig. 12A–E; Ng and Grinang 2018: fig. 5B–F, Ng and Ng 2019: fig. 9B–E, G, H, I–M). In addition, the distal corneous part of the G1 is almost straight in *G. sodalis* sp. nov. (Fig. 3B–D, F, H–K) but gently upcurved in *G. gracillimum* (see Ng and Ng 2019: fig. 9I–M). Compared to *G. bau*, which also has a more slender G1, *G. sodalis* sp. nov. has the distal part bent at an angle of about 45° along the longitudinal axis and the subdistal part of the outer margin is more angular and shelf-like (Figs 2H–K, 3B–D, F) (versus G1 bent at about 30° along longitudinal axis and subdistal part of outer margin is gradually sloping in *G. bau*; see Ng and Grinang 2004: fig. 9D, F).

The relatively longer fingers (distinctly longer than the palm) and the outer surface of the chela with fewer small granules in *G. sodalis* sp. nov. (Fig. 2A–D), differ from the condition in *G. katibas* and *G. larsi*, with the shorter fingers and the outer surface densely covered with small rounded granules (see Ng and Grinang 2018: figs 2D, 3A; Ng and Ng 2019: fig. 1C). The longer fingers of the chela most closely resemble those of *G. gracillimum* and *G. bau* (see Ng 1995: fig. 13A; Ng and Grinang 2004: fig. 8A; Ng 2015: fig. 14E, F). The male pleon of *G. sodalis* sp. nov. (Fig. 2G) is similar to that of *G. katibas* (see Ng and Ng 2019: fig. 8D), but this character is not reliable to differentiate taxa as it varies some degree in relative widths of the somites and convexity of the lateral margins of somite 6 (Ng and Ng 2019).

The male chela and G1 differences between *G. sodalis* sp. nov. and *G. spectrum* (from Brunei) are the same as for the Sarawakian *G. katibas*. *Geosesarma sodalis* sp. nov. differs markedly from the two species in this group from Indonesian Kalimantan, *G. ambawang* and *G. pontianak*, in possessing a G1 that is proportionately stouter and the subdistal part of the outer margin has a prominent right angled hump-like arch (see Ng 2015: figs 9D–G, 13D–H, J–M). The three species in this group from the eastern Malaysia state of Sabah, *G. sabanus*, *G. aurantium*, and *G. danumense* differ markedly from *G. sodalis* sp. nov. in that the corneous G1 distal part is longer and distinctly spatuliform in lateral view (Ng 1992, 1995, 2002; Ng and Ng 2018).

##### Biology.

Noteworthy is that *G. sodalis* sp. nov. was collected inside a cave where a cavernicolous species of gecarcinucid, *Arachnothelphusa sarang* Grinang & Ng, 2021, is present. Bukit Sarang is an isolated limestone outcrop with a complex of small caves, most of which probably have subterranean interconnections, and is part of the Tatau river basin in central Sarawak. The type specimen was obtained in moist areas several hundred meters from the cave entrance (RB Stuebing pers. comm.). Although more surveys in and around the Bukit Sarang were conducted in 2006 and more specimens of *A. sarang* were collected (Grinang and Ng 2021), no other specimens of *Geosesarma* were forthcoming.

*Geosesarma sodalis* sp. nov., however, does not have prominently elongated legs or reduced eyes typical of true troglobitic taxa, and must be treated as troglophile. It is probably more widespread outside the cave habitat. The site it was collected from is several hundred metres from the cave entrance and there was no light at all. The sympatric *Arachnothelphusa sarang* possesses some cave-dwelling characters-there is hardly any pigmentation on the body and legs and the pereopods are elongated, but the eyes are not reduced with the cornea still distinct, with Grinang and Ng (2021) treating it only as a troglophilic species.

No *Geosesarma* species had previously been recorded from caves, although one sesarmid genus *Karstarma* Davie & Ng, 2007, is known to live in or closely associated with limestone caves. *Karstarma* species are widely distributed in the Indo-West Pacific, with 18 recognised species (see Wowor and Ng 2018; Poupin et al. 2018; Ng 2020). Wowor and Ng (2018) recognised three species-groups in *Karstarma* and discussed the affinities of one of these groups with *Geosesarma*. They commented that the characters of some *Karstarma* species (e.g, *K. microphthalmus* (Naruse & Ng, 2007) and *K. malang* Wowor & Ng, 2018) are close to *Geosesarma*. Until the present discovery of *G. sodalis* sp. nov., no species of *Geosesarma* has previously been found in caves. *Geosesarma sodalis* sp. nov., however, has none of the morphological features associated with a cavernicolous lifestyle, e.g., reduced eyes and/or cornea and elongated pereopods. In any case, *G. sodalis* sp. nov. differs markedly from the group of *Karstarma* species highlighted by Wowor and Ng (2018) in its quadrate carapace, proportionately shorter ambulatory legs, and stouter G1, as well as its well-developed eyes with the large pigmented cornea.

Another species of sesarmid which was originally desrribed from near the entrance of a cave in Myanmar, *Pseudosesarma brehieri* Ng, 2018, is now known to normally live in mangrove habitats (Ng 2018; Schubart and Ng 2020).

## Supplementary Material

XML Treatment for Geosesarma

XML Treatment for Geosesarma sodalis
